# Effectiveness of virtual reality for functional disorders in cerebral palsy: an overview of systematic reviews and meta-analyses

**DOI:** 10.3389/fneur.2025.1582110

**Published:** 2025-07-30

**Authors:** Enhui Fang, Hui Guan, Binhong Du, Xuejun Ma, Lihong Ma

**Affiliations:** ^1^School of Rehabilitation Medicine, Shandong University of Traditional Chinese Medicine, Jinan, China; ^2^Department of Rehabilitation, Jinan Children's Hospital, Jinan, China

**Keywords:** virtual reality, cerebral palsy, neurorehabilitation, overview, systematic review

## Abstract

**Objective:**

Cerebral palsy (CP), a pediatric neuromotor disorder, profoundly impacts functional independence and participation. Virtual reality (VR) has developed as a potential neurorehabilitation tool, yet its therapeutic efficacy remains inconsistently validated. This overview aims to synthesize evidence from systematic reviews (SRs) and meta-analyses (MAs) to evaluate VR’s effectiveness in CP rehabilitation.

**Methods:**

Systematic searches across ten databases—Embase, Web of Science, Cochrane Library, PubMed, CINAHL, JBI, China National Knowledge Infrastructure (CNKI), China Science and Technology Journal Database (VIP), China Bio-Medical Literature Service System (Sino-Med), and Wanfang Database—identified SRs/MAs on VR for CP from inception to November 10, 2024. The duplicate rate of primary studies was assessed by calculating the corrected covered area (CCA) through the establishment of a literature overlap matrix. Methodological rigor, reporting quality, bias risk, and evidence quality were appraised using the Assessment of Multiple Systematic Reviews 2 (AMSTAR-2), the Preferred Reporting Items for Systematic Reviews and Meta-Analyses 2020 (PRISMA2020), the Risk of Bias in Systematic Reviews (ROBIS), and the Grading of Recommendations Assessment, Development and Evaluation (GRADE) tools, respectively.

**Results:**

Sixteen SRs/MAs (5 low quality, 11 very low quality, according to AMSTAR-2) were included. The CCA was calculated as 0.135, indicating a high degree of overlap. PRISMA 2020 compliance revealed incomplete reporting in 37% of items. ROBIS indicated low bias risk in 13 studies. GRADE assessments classified 58 outcomes: 9 moderate (15.5%), 21 low (36.2%), and 28 very low (48.3%) quality. VR demonstrated clinical potential for improving motor function and activities of daily living (ADL), particularly in younger children with higher intervention dosages. However, heterogeneity in outcome measures, CP subtypes, and VR protocols limited generalizability.

**Conclusion:**

VR shows potential in improving motor dysfunction and ADL in CP. However, the included SRs/MAs typically exhibited low methodological and evidence quality. Therefore, caution must be taken when interpreting these findings. Moreover, high-quality randomized controlled trials and standardized VR protocols are urgently needed to establish evidence-based guidelines for CP rehabilitation.

**Systematic review registration:**

https://www.crd.york.ac.uk/PROSPERO/view/CRD42024614631, CRD42024614631.

## 1 Introduction

Cerebral palsy (CP), a nonprogressive neurodevelopmental disorder originating from prenatal or perinatal brain insults, manifests as persistent motor and postural dysfunction with associated activity limitations (1). This central motor disorder is commonly comorbid with multisystem impairments, including sensory deficits, perceptual-cognitive dysfunction, communication-behavioral disorders, epilepsy, and secondary musculoskeletal pathologies (2). In the United States, large-scale surveys reported the prevalence of CP among children as 3.2 per 1,000 children aged 3–17 years, aligning with global estimates (3). Of note, the prevalence of pediatric physical disabilities overall, including conditions like cerebral palsy, varies by country, region, and population subgroup (4). Rehabilitation for children with CP aims to improve mobility, correct posture, and address abnormal movement patterns to enhance daily functioning (5). However, common barriers to adherence are fatigue, lack of motivation, and the repetitive or unengaging nature of traditional rehabilitation activities. Children are less likely to consistently participate in therapies that do not capture their interest or provide variety (6).

Virtual reality (VR) has developed as a promising neurorehabilitation tool for CP (7). VR is a three-dimensional, computer-generated virtual environment that supports work, education, entertainment, and health by simulating real-world or fictional settings and interactions (8). Currently, VR technologies are classified based on two primary dimensions: application type, which encompasses commercial systems versus rehabilitation-specific systems, and immersion level, ranging from non-immersive (screen-based interfaces) to semi-immersive (projection environments) and fully immersive configurations utilizing head-mounted displays (9). Notably, VR technologies can enhance emotionally relevant experiences and social interactions, offer safe, repeatable, and diversifiable interventions, and reduce anxiety levels in children (10). Neurophysiological studies reveal that when children display actions such as smiling, laughing, dancing, or screaming during virtual games, the bioelectrical signals in their brains reflect activation of the reward system, excitation of motor areas, and increased emotional and cognitive load. These changes are not only associated with the emotional experiences within the game but also linked to neuroplasticity in the brain (11). Furthermore, the VR training protocols, characterized by task-specific movement and adaptive difficulty modulation based on individualized capacity, enhance the transferability of motor skill acquisition to real-world functional task performance (12). VR systems also optimize rehabilitation dosing through patient-driven task selection, sustaining intrinsic motivation to achieve higher intervention intensity and frequency than traditional protocols (13). Moreover, the multisensory feedback mechanisms inherent in VR systems generate real-time performance metrics through audiovisual signals, enabling some degree of dynamic movement correction and contributing to improvements in motor skills (14). Collectively, these mechanisms address the International Classification of Functioning, Disability, and Health domains by mitigating environmental barriers and enhancing participation in educational and social contexts (15).

As evidence-based medicine has developed, researchers have performed an increasing number of systematic reviews (SRs) and meta-analyses (MAs) to assess the safety and effectiveness of VR for treating CP. However, due to differences in study design, limitations in sample size, and heterogeneity of outcomes, conflicting conclusions have emerged from various SRs/MAs, thereby compromising the reliability of the results. To address this issue, overviews of SRs use explicit and systematic methods to search for and identify multiple SRs on related research questions within the same topic area. Summarizing the evidence from multiple SRs allows for a more comprehensive analysis and summary of the findings, thereby providing higher quality evidence for clinical practice (16). This overview utilized the Assessment of Multiple Systematic Reviews 2 (AMSTAR-2) for methodological rigor evaluation (17), the Preferred Reporting Items for Systematic Reviews and Meta-Analyses 2020 (PRISMA2020) for reporting quality assessment (18), the Risk Of Bias In Systematic Reviews (ROBIS) for bias risk evaluation (19), and the Grading of Recommendations Assessment, Development and Evaluation (GRADE) for evidence quality grading (20). Specifically, the AMSTAR-2 tool focuses on a comprehensive evaluation of methodological quality in SRs/MAs, identifying critical design flaws (17). The PRISMA 2020 checklist evaluates the clarity, completeness, and transparency of SRs/MAs reporting through its standardized framework that spans from study design to result presentation, thereby helping to identify critical information omissions (18). The ROBIS tool specializes in assessing bias risks in SRs/MAs, particularly identifying factors across various domains that may compromise the reliability of conclusions (19). The GRADE approach systematically classifies evidence quality by appraising the quality of evidence based on key factors, enabling clinicians and policymakers to evaluate the credibility and applicability of research findings (20). Through this integrative approach, the purpose of this overview is to assess the methodological quality, reporting quality, risk of bias, and quality of evidence of SRs/MAs of VR for CP and to objectively and comprehensively evaluate the efficacy and safety of VR for CP, thereby providing an evidence-based basis for clinical decision-making.

## 2 Methods

The methodology of this overview follows the Cochrane Handbook, and its report is in line with the PRISMA 2020 checklist. This overview has been prospectively registered on PROSPERO (CRD42024614631).

### 2.1 Inclusion and exclusion criteria

This research incorporated SRs/MAs of randomized controlled trials (RCTs) that exclusively examined VR-based interventions for pediatric CP. Eligible studies were required to (1) have participants who were diagnosed with cerebral palsy and were aged ≤18 years; (2) compare VR monotherapy or VR-augmented conventional rehabilitation against control groups receiving conventional therapy or no intervention; and (3) report quantitative outcomes in at least one functional domain, specifically upper extremity motor function, gross motor capacity, balance, ambulation, or activities of daily living (ADL). Exclusion criteria comprised SRs/MAs incorporating non-RCT designs, studies involving non-CP populations, studies with non-extractable data or inaccessible full texts, and non-Chinese/English literature.

### 2.2 Search strategy

A comprehensive search for literature was executed across ten online databases: Embase, Web of Science, Cochrane Library, PubMed, CINAHL, JBI, China National Knowledge Infrastructure (CNKI), China Science and Technology Journal Database (VIP), China Bio-Medical Literature Service System (Sino-Med), and Wanfang Database, targeting SRs/MAs evaluating VR interventions for CP. The search strategy includes Chinese and English terms, combining MeSH/Emtree terms and free-text keywords. Chinese search terms included “脑瘫” (cerebral palsy), “脑性瘫痪” (cerebral paralysis), “虚拟现实” (virtual reality), “虚拟环境” (virtual environment), “虚拟康复” (virtual rehabilitation), “虚拟游戏” (virtual gaming), “虚拟治疗” (virtual therapy), “系统评价” (systematic review), “Meta分析” (meta-analysis), “荟萃分析” (pooled analysis), “系统综述” (systematic overview), and “元分析” (meta-synthesis). English terms comprised “cerebral palsy,” “virtual reality,” “virtual reality exposure therapy,” “meta-analysis as topic,” “meta-analysis,” and “systematic review.” The search encompassed all available records from each database’s inception through November 10, 2024. The reference list was also manually screened to ensure comprehensiveness. Two reviewers independently conducted the literature search, and the search results were cross-checked. Discrepancies were resolved through consensus discussions. If conflicts remained unresolved, a third reviewer made the final decision. The complete search syntax, with database-specific adaptations, is detailed in Supplementary Material 1.

### 2.3 Study selection and data extraction

Duplicates were eliminated after importing the retrieved SRs/MAs to EndNote 21. Two independent reviewers performed a primary screening by reading the title and abstract and a secondary screening by reading the full text. Consensus discussions were employed to resolve discrepancies, with unresolved conflicts adjudicated by a third reviewer. Two reviewers independently extracted data from the included literature and cross-checked the results for accuracy. Extracted data included authors, publication year, country, number of included studies, sample size, CP subtypes, VR protocols, comparator therapies, risk of bias assessment tools, evidence quality instruments, adverse events, and outcome measures.

### 2.4 Overlapping of studies

To evaluate the degree of overlap among primary studies included in multiple SRs/MAs, a literature overlap matrix was constructed to calculate the corrected covered area (CCA) (21). This metric accounts for redundant inclusions by using the formula CCA = (n − r)/(rc − r), where *n* represents the total number of primary studies across all SRs/MAs, r denotes the number of unique primary studies after deduplication, and c indicates the number of SRs/MAs included in the study. The CCA values were interpreted as follows: 0–5% (slight overlap), 6–10% (moderate overlap), 11–15% (high overlap), and >15% (very high overlap).

### 2.5 Assessment methods

In the PROSPERO-registered protocol of this overview, we initially planned to assess the quality of SRs/MAs exclusively using AMSTAR-2 and GRADE. However, during implementation, we additionally incorporated the PRISMA 2020 checklist and ROBIS tool. We made this decision because AMSTAR-2 and GRADE alone could not comprehensively evaluate reporting quality or risk of bias. PRISMA 2020 provides a more rigorous assessment of reporting transparency, complementing AMSTAR-2’s methodological focus. ROBIS systematically addresses potential biases that neither AMSTAR-2 nor GRADE explicitly covers. This expansion aimed to enhance the robustness of our quality assessment framework. The included SRs/MAs were assessed independently by two reviewers, and the results were cross-checked. Consensus discussions were utilized to resolve discrepancies. A third reviewer then adjudicated any unresolved conflicts.

#### 2.5.1 Methodological rigor assessment

We evaluated the methodological rigor of the included SRs/MAs using the AMSTAR-2, which comprises 16 appraisal items. Each item was rated as “Yes,” “No,” or “Partial Yes.” The final quality categorization was determined by 7 critical items (items 2, 4, 7, 9, 11, 13, and 15) according to the AMSTAR-2 criteria: High (no non-critical deficiencies or only one non-critical deficiency), Moderate (more than one non-critical deficiency), Low (one critical deficiency with or without non-critical deficiencies), and Critically Low (more than one critical deficiency with or without non-critical deficiencies) (17).

#### 2.5.2 Report quality assessment

The PRISMA 2020 is based on PRISMA 2009 and has been revised through a rigorous literature review, expert consultation, and consensus conference, and its methodology is scientifically rigorous and highly credible. The PRISMA 2020 has a broader scope of application, primarily for systematic reviews assessing the effectiveness of health interventions, including those with and without meta-analyses. The completeness of reporting was assessed using the PRISMA 2020 checklist, comprising 27 reporting items across seven methodological domains. Each item was scored using a three-tiered classification system: full compliance, partial compliance, and non-compliance (18).

#### 2.5.3 Risk of bias assessment

The ROBIS tool employs a three-phase structured evaluation protocol: Phase 1 (Relevance Assessment) examines the congruence between the review’s objectives and the research questions addressed. Phase 2 (Risk of Bias Determination) systematically evaluates four methodological domains: (1) eligibility criteria: whether predefined inclusion/exclusion criteria align logically with the research question; (2) search and selection: adequacy of literature retrieval strategies to minimize missed eligible studies; (3) data extraction and quality appraisal: rigor in data collection processes and validity of critical appraisal tools; (4) synthesis and presentation: appropriateness of statistical methods for data integration and result reporting. Phase 3 (final risk judgment): the overall bias risk is adjudicated by evaluating whether reviewers appropriately addressed the identified biases from Phase 2, critically appraised the relevance of the included studies to the research question, and avoided overemphasis on statistically significant outcomes. Each domain receives a categorical rating: high risk, low risk, or unclear risk (19).

#### 2.5.4 Evidence quality assessment

The GRADE approach evaluates the quality of evidence across five dimensions: risk of bias, inconsistency, indirectness, imprecision, and publication bias. Specifically, risk of bias refers to the extent of systematic errors introduced during study design or execution, which may compromise the validity and reliability of findings. Consistency reflects the similarity of effect estimates across studies; significant variability between estimates indicates substantial inconsistency. Indirectness describes the applicability of study populations, interventions, or outcomes to the target clinical context; evidence is deemed indirect when direct extrapolation is unjustified. Imprecision arises when confidence intervals for effect estimates are extensive, reflecting substantial uncertainty. Publication Bias occurs when the likelihood of a study’s publication is influenced by statistical significance or effect magnitude, potentially skewing synthesized evidence. Final evidence quality is classified into four tiers: High (no downgrading), Moderate (1-level downgrade), Low (2-level downgrade), and Very Low (≥3-level downgrade), based on the cumulative impact of these dimensions (20).

## 3 Results

### 3.1 Search results

**Figure 1:**
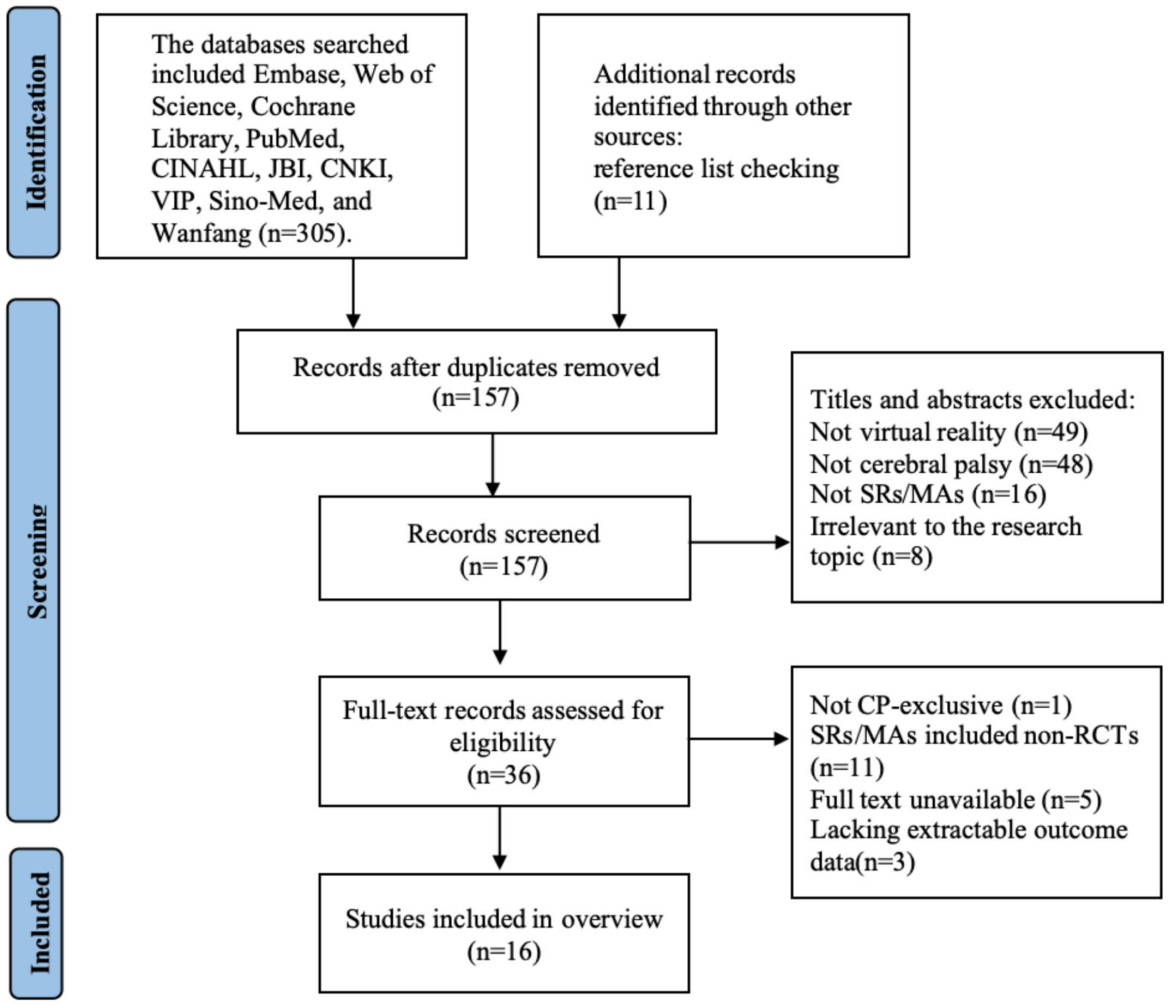
Literature screening flow chart.

A total of 316 records were identified. During the systematic screening, 159 duplicate records were excluded, yielding 157 records for title/abstract screening. Applying predefined inclusion criteria, exclusions at this stage included non-virtual reality interventions (*n* = 49), non-cerebral palsy populations (*n* = 48), non-SRs/MAs (*n* = 16), and irrelevance to the research topic (*n* = 8), resulting in 36 records proceeding to full-text retrieval. Critical appraisal of full texts led to further exclusions: one study for mixed population inclusion, 11 SRs/MAs containing non-RCTs, five studies with inaccessible full texts, and three studies lacking extractable outcome data. Ultimately, 16 studies met eligibility criteria for final inclusion. The selection procedure is presented in Figure 1.

### 3.2 Literature characteristics

The 16 SRs/MAs comprised 13 English-language and 3 Chinese-language publications. Five studies (22–26) were classified as meta-analyses, two studies (27, 28) were classified as systematic reviews, and nine studies (29–37) were classified as a combination of systematic review and meta-analysis. Two SRs/MAs were specific to children with spastic CP, while the remaining encompassed all CP subtypes. The quantity of original RCTs per SR/MA ranged from 9 to 38, with aggregate sample sizes spanning 276 to 1,223 pediatric participants. The included SRs/MAs demonstrated variable focus across functional outcome domains: upper extremity motor function was evaluated in 7 SRs/MAs (28–30, 33, 35–37); gross motor function in 9 SRs/MAs (22–24, 26, 27, 30, 31, 35, 37); balance function in 9 SRs/MAs (22, 23, 25, 27, 28, 31, 32, 35, 37); ambulation function in 5 SRs/MAs (22, 23, 27–29); and ADL function in 7 SRs/MAs (23, 26, 31, 33–35, 37). The literature characteristics are detailed in Table 1.

**Table 1:** Characteristics of the included SRs/MAs.

Included studies	Country	No. of RCTs	Participants	Disease type	Experimental intervention	Control intervention	Risk assessment tools	Quality of evidence evaluation tool	Adverse effects	Outcomes
Lin et al. (2019) (22)	China	9	281	All types	VR	CR	Cochrane Risk of Bias tool	NR	NR	②③④
Luo et al. (2023) (23)	China	18	662	All types	VR	CR	Cochrane Risk of Bias tool	NR	NR	②③④⑤
Han et al. (2020) (27)	China	14	432	All types	VR + CR	CR	Cochrane Risk of Bias tool	NR	NR	②③④
Chen et al. (2018) (29)	United States	19	514	All types	VR	CR, NI	PEDro scale	NR	NR	①④
Ren et al. (2019) (24)	China	9	376	All types	VRGs, VRGs+CR	CR	PEDro scale	NR	NR	②
Wu et al. (2019) (25)	China	11	448	All types	VR, VR + CR	CR	PEDro scale	NR	NR	③
Fandim et al. (2021) (28)	Brazil	38	1,223	All types	VR, VR + CR	CR, NI	Cochrane Risk of Bias tool	GRADE	No significant	①③④
Abdelhaleem et al. (2022) (30)	Egypt	19	645	All types	VR, VR + CR	CR	Cochrane Risk of Bias tool	NR	No significant	①②
Liu et al. (2022) (31)	China	16	513	spasticity	VR, VR + CR	CR	Cochrane Risk of Bias tool	NR	NR	②③⑤
Liu et al. (2022) (32)	China	18	474	All types	VR, VR + CR	CR	PEDro scale	NR	NR	③
Montoro et al. (2022) (33)	Spain	9	276	All types	NWT, NWT + CR	CR	Cochrane Risk of Bias tool	GRADE	NR	①⑤
Han et al. (2023) (34)	South Korea	11	442	All types	VR	CR, NI	Cochrane Risk of Bias tool	NR	NR	⑤
Wang et al. (2023) (35)	China	21	779	spasticity	VR	CR	PEDro scale	NR	NR	①②③⑤
Burin et al. (2024) (36)	France	22	746	All types	VR, VR + CR	CR, NI	PEDro scale	GRADE	NR	①
Komariah et al. (2024) (37)	Indonesia	19	894	All types	VR	CR, NI	Cochrane Risk of Bias tool	NR	NR	①②③⑤
Zhang et al. (2022) (26)	China	17	853	All types	VR	CR	Cochrane Risk of Bias tool	GRADE	NR	②⑤

VR, virtual reality; CP, cerebral palsy; NWT, nintendo^®^ Wii; VRGs, virtual Reality Games; CR, conventional rehabilitation; NI, no intervention; NR, not reported; ① Upper limb function; ② Gross motor function; ③ Balance; ④ Ambulation; ⑤ Activities of Daily Living.

### 3.3 Overlapping of studies

This study included a total of 16 SRs/MAs, encompassing 270 primary studies before deduplication and 89 unique studies after deduplication. The CCA was calculated as 0.135, indicating a high degree of overlap (Supplementary Materials 2 contains a spreadsheet used to calculate CCA). As the aim of this study was to summarize outcome measures rather than perform quantitative synthesis, no statistical methods were applied to address overlapping studies.

### 3.4 Results of methodological rigor assessment

The AMSTAR-2 assessment revealed significant methodological limitations across the 16 SRs/MAs, with 5 rated as low quality and 11 as very low quality. Critical domain deficiencies included protocol registration in only 7 SRs/MAs (26, 28, 33–37), comprehensive search strategies in 2 (26, 33), provision of excluded study lists with justifications in 1 (29), and failure to address risk of bias in 5 (24, 25, 29, 31, 32) or publication bias impacts in 6 (22, 23, 28, 30, 32, 37). While all SRs/MAs utilized validated tools for bias assessment and appropriate synthesis methods, deficiencies across ≥1 critical domain and multiple non-critical items collectively downgraded methodological rigor. The AMSTAR-2 evaluation results are presented in Table 2.

**Table 2:** Methodological rigor of included SRs/MAs.

Included studies	AMSTAR2																Overall quality
	Q1	Q2*	Q3	Q4*	Q5	Q6	Q7*	Q8	Q9*	Q10	Q11*	Q12	Q13*	Q14	Q15*	Q16	
Lin et al. (2019) (22)	Y	N	N	PY	Y	Y	N	Y	Y	N	Y	Y	Y	Y	N	N	CL
Luo et al. (2023) (23)	Y	N	N	PY	Y	N	N	Y	Y	N	Y	Y	Y	Y	N	N	CL
Han et al. (2020) (27)	Y	N	N	PY	Y	N	N	Y	Y	N	Y	N	Y	N	Y	N	CL
Chen et al. (2018) (29)	Y	N	Y	PY	N	Y	Y	Y	Y	N	Y	N	N	Y	Y	Y	CL
Ren et al. (2019) (24)	Y	N	N	PY	N	Y	N	Y	Y	N	Y	Y	N	Y	Y	Y	CL
Wu et al. (2019) (25)	Y	N	N	PY	Y	Y	N	Y	Y	N	Y	N	N	Y	Y	Y	CL
Fandim et al. (2021) (28)	Y	Y	N	PY	Y	N	N	Y	Y	N	Y	Y	Y	N	N	Y	CL
Abdelhaleem et al. (2022) (30)	Y	N	N	PY	Y	Y	N	Y	Y	N	Y	N	Y	N	N	Y	CL
Liu et al. (2022) (31)	Y	N	N	PY	Y	Y	N	Y	Y	N	Y	Y	N	Y	Y	N	CL
Liu et al. (2022) (32)	Y	N	N	PY	Y	Y	N	Y	Y	N	Y	N	N	Y	N	Y	CL
Montoro et al. (2022) (33)	Y	Y	N	Y	Y	Y	N	Y	Y	N	Y	N	Y	Y	Y	Y	L
Han et al. (2023) (34)	Y	Y	N	PY	Y	Y	N	Y	Y	N	Y	Y	Y	Y	Y	Y	L
Wang et al. (2023) (35)	Y	Y	N	PY	Y	N	N	Y	Y	N	Y	Y	Y	Y	Y	N	L
Burin et al. (2024) (36)	Y	Y	N	PY	N	Y	N	Y	Y	Y	Y	N	Y	Y	Y	Y	L
Komariah et al. (2024) (37)	Y	Y	Y	PY	Y	Y	N	Y	Y	N	Y	N	Y	Y	N	N	CL
Zhang et al. (2022) (26)	Y	Y	N	Y	Y	Y	N	Y	Y	N	Y	N	Y	Y	Y	Y	L
Y + PY/total (%)	100	43.8	12.5	100	81.3	75	6.25	100	100	6.25	100	43.8	68.8	81.3	62.5	62.5	

*indicates key items. Item 1: The review’s research questions and inclusion criteria explicitly incorporated the PICO components. Item 2: The review report stated that methods were established a priori and justified any significant deviations from the registered protocol. Item 3: Authors provided a rationale for their selection of eligible study designs. Item 4: A systematic search strategy was employed, including multiple databases and grey literature without language restrictions. Item 5: Study selection was performed independently by at least two reviewers with conflict resolution procedures. Item 6: Data extraction was conducted in duplicate to ensure accuracy. Item 7: A list of excluded studies with justification was provided. Item 8: Included studies were described in sufficient detail. Item 9: Standardized tools were used to evaluate bias in individual studies. Item 10: Sources of funding for included studies were reported. Item 11: When applicable, appropriate statistical methods were used for meta-analysis with justification. Item 12: The influence of individual study bias on meta-analysis/evidence synthesis results was assessed. Item 13: Review authors discussed how risk of bias in primary studies affected their conclusions. Item 14: Observed heterogeneity was explored and discussed. Item 15: For quantitative synthesis, publication bias was assessed and its potential impact addressed. Item 16: All review-related funding and potential conflicts of interest were disclosed. H: indicates high quality; M: indicates moderate quality; L: indicates low quality; and CL: indicates critically low quality. N, no; PY, partial yes; Y, yes.

### 3.5 Results of reporting quality assessment

The PRISMA 2020 checklist identified that 17 of 27 items were fully reported, while 10 were inadequately reported. Methodological deficiencies included (1) omission of study selection processes in 2 SRs/MAs (24, 29); (2) undocumented data extraction protocols in 3 SRs/MAs (23, 27, 35); and (3) failure to assess cross-study risk of bias in 5 SRs/MAs (22, 23, 30, 32, 37). Only 25% of SRs/MAs explicitly described evidence synthesis methodologies (26, 28, 33, 36). In results reporting, 4 SRs/MAs neglected publication bias analysis (23, 30, 32, 37), and only 25% implemented evidence quality grading (26, 28, 33, 36). Transparency shortcomings comprised protocol registration in 7 SRs/MAs (26, 28, 33–37), undisclosed funding sources in 3 (31, 35, 37), omitted conflict-of-interest statements in 3 (22, 23, 27), and publicly accessible data in only 3 SRs/MAs (26, 33, 34). The results of the PRISMA 2020 are presented in Table 3.

**Table 3:** Results of the PRISMA checklist for the included SRs/MAs.

Section/topic	Items	Complete report	Partial report	Not reported	Compliance (%)
Title	Q1. Title	16 (22–37)	0	0	100
Abstract	Q2. Structured summary	16 (22–37)	0	0	100
Introduction	Q3. Rationale	16 (22–37)	0	0	100
	Q4. Objectives	16 (22–37)	0	0	100
Methods	Q5. Eligibility criteria	16 (22–37)	0	0	100
	Q6. Information sources	16 (22–37)	0	0	100
	Q7. Search	4 (28, 32, 36, 37)	12 (22–27, 29–31, 33–35)	0	100
	Q8. Study selection	13 (22–24, 27–34, 36–37)	1 (35)	2 (25–26)	87.5
	Q9. Data collection process	13 (22, 25–33, 35–37)	0	3 (23–24, 34)	81.25
	Q10. Data items	15 (23–37)	1 (22)	0	100
	Q11. Risk of bias in individual studies	14 (22–23, 26–37)	2 (24–25)	0	100
	Q12. Summary measures	16 (22–37)	0	0	100
	Q13. Synthesis of results	16 (22–37)	0	0	100
	Q14. Risk of bias across studies	11 (24–28, 30, 32–35, 37)	0	5 (22–23, 29, 31, 36)	68.75
	Q15. Summary of evidence	4 (28, 32, 35, 37)	0	12 (22–27, 29–31, 33–34, 36)	25
Results	Q16. Study selection	16 (22–37)	0	0	100
	Q17. Study characteristics	16 (22–37)	0	0	100
	Q18. Risk of bias within studies	15 (22–24, 26–37)	1 (25)	0	100
	Q19. Results of individual studies	16 (22–37)	0	0	100
	Q20. Synthesis of results	16 (22–37)	0	0	100
	Q21. Risk of bias across studies	12 (22, 24–28, 30, 32–35, 37)	0	4 (23, 29, 31, 36)	75
	Q22. Summary of evidence	4 (28, 32, 35, 37)	0	12 (22–27, 29–31, 33–34, 36)	25
Discussion	Q23. Discussion	16 (22–37)	0	0	100
Others	Q24. Protocol and registration	7 (28, 32–37)	0	9 (22–27, 29–31)	43.75
	Q25. Funding	13 (22–29, 31–33, 35, 37)	0	3 (30, 34, 36)	81.25
	Q26. Conflict of interest	13 (25–37)	0	3 (22–24)	81.25
	Q27. Data availability	3 (32–33, 37)	0	13 (22–31, 34–36)	18.75

### 3.6 Results of risk of bias assessment

During phase 1, all 16 SRs/MAs were classified as low risk of bias, confirming alignment with the research objectives. In phase 2, domain 1 achieved universal low-risk compliance. In contrast, domain 2 revealed critical deficiencies, with only 3 SRs/MAs meeting low-risk standards (26–28). One SR/MA could not be conclusively rated due to ambiguous search syntax and unspecified screening personnel (29), while 12 SRs/MAs exhibited high-risk bias from non-exhaustive database searches, notably omitting clinical trial registries. In domain 3, 3 SRs/MAs were unrated due to undocumented extractor roles (27–29), and 13 SRs/MAs maintained low-risk ratings. Domain 4 identified instability risks in 2 SRs/MAs due to absent sensitivity analyses (30, 36), while 14 SRs/MAs retained low-risk status. In phase 3, 3 SRs/MAs were escalated to high-risk classification for insufficient bias rationale (30, 32, 36), and the remaining 13 SRs/MAs preserved low-risk ratings. The results of the bias risk evaluation are presented in Table 4.

**Table 4:** Risk of bias of the included SRs/MAs.

Included studies	Phase 1	Phase 2				Phase 3
	Assessing relevance	Domain 1: study eligibility criteria	Domain 2: identification and selection of studies	Domain 3: collection and study appraisal	Domain 4: synthesis and findings	Risk of bias in the review
Lin et al. (2019) (22)	Low risk	Low risk	High risk	Low risk	Low risk	Low risk
Luo et al. (2023) (23)	Low risk	Low risk	High risk	Low risk	Low risk	Low risk
Han et al. (2020) (27)	Low risk	Low risk	Low risk	Undetected risk	Low risk	Low risk
Chen et al. (2018) (29)	Low risk	Low risk	Undetected risk	Undetected risk	Low risk	Low risk
Ren et al. (2019) (24)	Low risk	Low risk	High risk	Low risk	Low risk	Low risk
Wu et al. (2019) (25)	Low risk	Low risk	High risk	Low risk	Low risk	Low risk
Fandim et al. (2021) (28)	Low risk	Low risk	Low risk	Undetected risk	Low risk	Low risk
Abdelhaleem et al. (2022) (30)	Low risk	Low risk	High risk	Low risk	High risk	High risk
Liu et al. (2022) (31)	Low risk	Low risk	High risk	Low risk	Low risk	Low risk
Liu et al. (2022) (32)	Low risk	Low risk	High risk	Low risk	Low risk	High risk
Montoro et al. (2022) (33)	Low risk	Low risk	High risk	Low risk	Low risk	Low risk
Han et al. (2023) (34)	Low risk	Low risk	High risk	Low risk	Low risk	Low risk
Wang et al. (2023) (35)	Low risk	Low risk	High risk	Low risk	Low risk	Low risk
Burin et al. (2024) (36)	Low risk	Low risk	High risk	Low risk	High risk	High risk
Komariah et al. (2024) (37)	Low risk	Low risk	High risk	Low risk	Low risk	Low risk
Zhang et al. (2022) (26)	Low risk	Low risk	Low risk	Low risk	Low risk	Low risk

### 3.7 Results of evidence quality assessment

The GRADE evidence quality assessment of the five outcome indicators identified 58 evidence bodies, with only 9 (15.5%) classified as moderate quality, while 21 (36.2%) and 28 (48.3%) were categorized as low and very low quality, respectively.

#### 3.7.1 Improvement of upper extremity motor function

VR interventions were evaluated for upper limb motor function among children with CP by seven SRs/MAs (28–30, 33, 35–37). The results of the GRADE evaluations for upper extremity motor function are shown in Table 5.

**Table 5:** The results of the GRADE evaluations for upper extremity motor function.

Outcomes and Included studies	EG vs CG	No. of RCTs	Participants	Bias risk	Inconsistency	Indirectness	Imprecision	Publication bias	Relative effect	95% CI	Quality
Upper extremity motor function											
Chen et al. (2018) (29)	VR vs. CR/NI	13	396	-1	0	0	-1	-1	Cohen’s d = 0.84	0.39–1.28	Very low
Fandim et al. (2021) (28)	VR + CR vs. CR	11	390	-1	−2	0	−1	−1	SMD = 1.06	0.42–1.69	Very low
Fandim et al. (2021) (28)	VR vs. CR	4	148	−1	−2	0	−1	−1	SMD = 0.48	−0.47 − 1.43	Very low
Fandim et al. (2021) (28)	VR vs. NI	2	115	-1	−2	0	−1	−1	SMD = 0.64	−0.40-1.67	Very low
Abdelhaleem et al. (2022) (30)	VR/VR + CR vs. CR	6	255	−2	−2	0	−1	−1	SMD = 0.75	0.02–1.51	Very low
Montoro et al. (2022) (33)	NWT + CR vs. CR	2	48	−1	0	0	−1	−1	SMD = 0.73	0.16–1.3	Very low
Montoro et al. (2022) (33)	NWT vs. CR	1	40	−1	0	0	−1	−1	SMD = 3.12	1.53–4.7	Very low
Montoro et al. (2022) (33)	NWT + CR vs. CR	2	68	−1	0	0	−1	−1	SMD = −0.05	−0.51-0.41	Very low
Montoro et al. (2022) (33)	NWT vs. CR	1	30	−1	0	0	−1	−1	SMD = -0.12	−0.84-0.6	Very low
Montoro et al. (2022) (33)	NWT + CR vs. CR	3	105	−1	0	0	−1	0	SMD = -0.28	−0.67-0.1	Low
Wang et al. (2023) (35)	VR vs. CR	3	113	−1	0	0	−1	0	MD = 6.24	4.87–7.60	Low
Wang et al. (2023) (35)	VR vs. CR	3	100	−1	0	0	−1	0	MD = 4.24	1.43–7.06	Low
Wang et al. (2023) (35)	VR vs. CR	3	194	−1	−1	0	−1	0	MD = −2.20	−8.22–3.81	Very low
Burin et al. (2024) (36)	VR + CR vs. CR	15	479	−1	−1	0	0	0	SMD = 0.65	0.19–1.11	Low
Burin et al. (2024) (36)	VR vs. CR	5	92	−1	−1	0	−1	0	SMD = 0.74	−0.32 − 1.81	Very low
Burin et al. (2024) (36)	VR vs. NI	2	131	-1	−2	0	−1	0	SMD = 1.61	−1.34–4.55	Very low
Komariah et al. (2024) (37)	VR vs. CR/NI	2	60	0	0	0	−1	0	MD = −0.50	−0.81 – −0.19	Moderate

##### 3.7.1.1 Assessment tools

Significant improvements in upper limb function were observed using the Childhood Health Assessment Questionnaire (moderate evidence) and Peabody Developmental Motor Scales-2/Quality of Upper Extremity Skills Test (low-to-very-low evidence) (33, 35, 37). However, no significant differences were found in the Jebsen-Taylor Hand Function Test between VR and conventional rehabilitation (very low evidence) (33).

##### 3.7.1.2 Intervention types

VR combined with conventional rehabilitation showed superior efficacy compared to conventional therapy alone in improving upper limb coordination (low-to-very-low evidence) (28, 36). In contrast, isolated VR training demonstrated conflicting results: while Montoro et al. reported significant benefits in fine motor coordination (very low evidence) (33), Abdelhaleem et al. found improvements only with combined interventions (very low evidence) (30), highlighting potential synergies between modalities.

##### 3.7.1.3 Subgroup analyses

A significant negative correlation emerged between participant age and effect size, with younger children achieving better outcomes, alongside a positive correlation between higher daily VR dosage and functional gains (29). Regarding system design, engineer-developed VR systems demonstrated superior therapeutic effects compared to commercial systems (moderate evidence) (29). Although both commercial video games and rehabilitation-specific systems outperformed conventional therapy (low evidence), no statistically significant difference was observed between these two VR modalities (36). It is important to note that the results of the subgroup analyses described above are based on a limited number of SRs/MAs. Therefore, the results should be interpreted with caution.

#### 3.7.2 Improvement of gross motor function

Gross motor function refers to the ability to coordinate the movement of large muscles, strengthen the core body position, and maintain balance during movement and postural changes (e.g., standing on one leg, walking, running, or jumping) (38–40). It lays the foundation for participation in physical activities, sports, and daily tasks and is linked to cognitive and social development (41). VR interventions were evaluated for gross motor function among children with CP by nine SRs/MAs (22–24, 26, 27, 30, 31, 35, 37). The results of the GRADE evaluations for gross motor function are shown in Table 6.

**Table 6:** The results of the GRADE evaluations for gross motor function.

Outcomes and included studies	EG vs CG	No. of RCTs	Participants	Bias risk	Inconsistency	Indirectness	Imprecision	Publication bias	Relative effect	95% CI	Quality
Gross motor function											
Lin et al. (2019) (22)	VR vs. CR	8	248	−1	0	0	−1	0	MD = 5.57	3.76—7.38	Low
Luo et al. (2023) (23)	VR vs. CR	9	402	−1	0	0	0	−1	SMD = 0.42	0.09—0.76	Low
Han et al. (2020) (27)	VR + CR vs. CR	8	596	−1	0	0	0	0	WMD = 5.55	3.48–7.61	Moderate
Ren et al. (2019) (24)	VR/VR + CR vs. CR	9	459	−1	0	0	0	0	SMD = 0.23	0.04–0.41	Moderate
Abdelhaleem et al. (2022) (30)	VR/VR + CR vs. CR	14	363	−2	0	0	−1	−1	SMD = 0.15	0.09–0.40	Very low
Liu et al. (2022) (31)	VR/VR + CR vs. CR	7	236	−1	0	0	−1	0	SMD = 0.60	0.34–0.87	Low
Wang et al. (2023) (35)	VR vs. CR	9	285	−1	0	0	−1	0	MD = 4.99	1.65–8.33	Low
Komariah et al. (2024) (37)	VR vs. CR/NI	6	154	0	0	0	−1	0	MD = 3.73	1.67–5.79	Moderate
Zhang et al. (2022) (26)	VR vs. CR	4	201	−1	0	0	−1	0	Hedges’ g = 0.71	0.43–0.99	Low

##### 3.7.2.1 Assessment tools

Of these, eight SRs/MAs employing the Gross Motor Function Measure (GMFM) demonstrated that VR interventions (either standalone or combined with conventional rehabilitation) significantly improved gross motor function versus conventional therapy or no intervention (moderate-to-very-low evidence) (22–24, 26, 27, 31, 35, 37). Specifically, VR showed superior efficacy in enhancing standing (GMFM-D) and walking/running/jumping (GMFM-E) compared to conventional rehabilitation (low evidence) (22). Notably, while significant improvements were observed in GMFM-88 scores across studies, no significant changes were detected in GMFM-66 scores (23, 27). Furthermore, Abdelhaleem et al. reported VR-induced enhancements in gross motor coordination (low evidence) (30).

##### 3.7.2.2 Subgroup analyses

Regarding the level of immersion, semi-immersive systems exhibited greater therapeutic effects than non-immersive or fully immersive systems (37). For intervention parameters, optimal outcomes were achieved with sessions lasting 17–40 min, delivered ≥5 times weekly, over >12 weeks, and cumulatively exceeding 1,000 min—aligning with evidence linking higher frequency and prolonged duration to enhanced motor adaptation (24, 25, 28, 31). Notably, VR demonstrated significant gross motor improvements exclusively in children with hemiplegia (31). The subgroup analysis results reported above were based on a limited number of SRs/MAs, which may restrict the generalizability and external validity of the findings. Thus, these results should be interpreted with caution.

#### 3.7.3 Improvement of balance

VR interventions were evaluated for balance function among children with CP by nine SRs/MAs (22, 23, 25, 27, 28, 31, 32, 35, 37). The results of the GRADE evaluations for balance function are shown in Table 7.

**Table 7:** The results of the GRADE evaluations for balance function.

Outcomes and included studies	EG vs CG	No. of RCTs	Participants	Bias risk	Inconsistency	Indirectness	Imprecision	Publication bias	Relative effect	95% CI	Quality
Balance											
Lin et al. (2019) (22)	VR vs. CR	3	64	−1	0	0	−1	0	MD = 4.63	2.88—6.37	Low
Lin et al. (2019) (22)	VR vs. CR	2	60	−1	−2	0	−1	0	MD = -1.09	−5.55—1.75	Very low
Luo et al. (2023) (23)	VR vs. CR	10	345	−1	O	0	−1	−1	SMD = 0.82	0.45—1.19	Very low
Han et al. (2020) (27)	VR + CR vs. CR	6	156	−1	0	0	−1	0	WMD = 2.57	1.75–3.38	Low
Wu et al. (2019) (25)	VR/VR + CR vs. CR	14	448	−1	0	0	0	0	SMD = 0.29	0.10–0.48	Moderate
Fandim et al. (2021) (28)	VR vs. CR	3	112	−1	−1	0	−1	−1	SMD = 1.43	0.61–2.24	Very low
Fandim et al. (2021) (28)	VR + CR vs. CR	6	124	−1	−1	0	−1	−1	SMD = 0.43	−0.11-0.97	Very low
Liu et al. (2022) (31)	VR/VR + CR vs. CR	6	126	−1	0	0	−1	0	MD = 2.06	1.15–2.97	Low
Liu et al. (2022) (31)	VR/VR + CR vs. CR	3	123	−1	−1	0	−1	0	MD = 3.66	0.29–7.02	Very low
Liu et al. (2022) (32)	VR/VR + CR vs. CR	16	470	−1	0	0	0	−1	SMD = 0.47	0.28–0.66	Low
Wang et al. (2023) (35)	VR vs. CR	6	126	−1	0	0	−1	0	MD = 2.09	1.18–3.00	Low
Wang et al. (2023) (35)	VR vs. CR	3	123	−1	−1	0	−1	0	MD = 2.43	0.32–4.53	Very low
Komariah et al. (2024) (37)	VR vs. CR/NI	8	206	0	0	0	−1	0	MD = 2.71	1.95–3.48	Moderate

##### 3.7.3.1 Assessment tools

Significant improvements were observed using the Berg Balance Scale and Pediatric Balance Scale when combining VR with conventional rehabilitation (low-to-very-low evidence) (27, 31, 35). However, no significant differences were detected in Timed Up and Go Test scores between VR and conventional therapy alone (very low evidence) (22).

##### 3.7.3.2 Intervention types

Isolated VR training showed superior efficacy compared to conventional rehabilitation in two studies (very low evidence) (28, 32). In contrast, combined VR-conventional rehabilitation demonstrated consistent benefits across studies (low evidence) (32), indicating potential synergistic effects of multimodal interventions.

##### 3.7.3.3 Subgroup analyses

Regarding immersion levels, semi-immersive systems significantly outperformed non-immersive systems in balance improvement (moderate evidence) (37), likely due to enhanced spatial awareness and proprioceptive feedback. For intervention parameters, longer intervention duration (≥12 weeks) enhanced therapeutic effects (very low evidence) (23). However, no significant associations were found between balance outcomes and session frequency/duration or total intervention time (moderate evidence) (25). Notably, the subgroup analysis results shown above were generated from a limited number of SRs/MAs, and they should be interpreted with caution.

#### 3.7.4 Improvement of ambulation

VR interventions were evaluated for ambulation function among children with CP by five SRs/MAs (22, 23, 27–29). The results of the GRADE evaluations for ambulation function are shown in Table 8.

**Table 8:** The results of the GRADE evaluations for ambulation function and ADL.

Outcomes and Included studies	EG vs CG	No. of RCTs	Participants	Bias risk	Inconsistency	Indirectness	Imprecision	Publication bias	Relative effect	95% CI	Quality
Ambulation											
Lin et al. (2019) (22)	VR vs. CR	2	48	−1	0	0	−1	0	MD = 0.18	−0.11-0.47	Low
Luo et al. (2023) (23)	VR vs. CR	3	78	−1	0	0	−1	−1	MD = 0.14	0.01–0.27	Very low
Luo et al. (2023) (23)	VR vs. CR	2	36	−1	−1	0	−1	−1	MD = 7.51	−26.20-41.23	Very low
Han et al. (2020) (27)	VR + CR vs. CR	4	104	0	0	0	−1	0	WMD = 0.16	0.04–0.27	Moderate
Chen et al. (2018) (29)	VR vs. CR/NI	8	282	−1	0	0	−1	−1	Cohen’s d = 0.76	0.35–1.16	Very low
Fandim et al. (2021) (28)	VR vs. CR	2	110	−1	0	0	−1	−1	MD = 0.33	−0.09-0.75	Very low
Fandim et al. (2021) (28)	VR + CR vs. CR	5	123	−1	0	0	−1	−1	SMD = -0.08	−0.45-0.29	Very low
Fandim et al. (2021) (28)	VR vs. NI	2	129	−1	0	0	−1	−1	SMD = 0.67	0.13–1.21	Very low
Activities of daily living											
Luo et al. (2023) (23)	VR vs. CR	3	128	−1	0	0	−1	−1	MD = -0.02	−3.89-3.84	Very low
Liu et al. (2022) (31)	VR/VR + CR vs. CR	6	250	−1	0	0	−1	0	SMD = 0.55	0.30–0.81	Low
Montoro et al.(2022) (33)	NWT vs. CR	1	30	−1	0	0	−1	−1	SMD = 0.82	0.07–1.56	Very low
Montoro et al.(2022) (33)	NWT + CR vs. CR	3	72	−1	0	0	−1	0	SMD = 0.43	−0.04-0.91	Low
Han et al. (2023) (34)	VR vs. CR/NI	11	442	−1	0	0	0	0	SMD = 0.37	0.17–0.57	Moderate
Wang et al. (2023) (35)	VR vs. CR	3	108	−1	0	0	−1	0	MD = 7.55	3.35–11.76	Low
Wang et al. (2023) (35)	VR vs. CR	2	74	−1	0	0	−1	0	MD = 4.67	2.01–7.33	Low
Wang et al. (2023) (35)	VR vs. CR	2	126	−1	0	0	−1	0	MD = 1.35	0.78–1.93	Low
Komariah et al. (2024) (37)	VR vs. CR/NI	5	168	0	−1	0	−1	0	MD = 10.05	2.89–17.22	Low
Zhang et al. (2022) (26)	VR vs. CR	11	566	−1	0	0	0	0	Hedges’ g = 0.31	0.10–0.51	Moderate

##### 3.7.4.1 Assessment tools

Meta-analyses of the 10-Meter Walk Test revealed conflicting evidence: while two SRs/MAs reported significant walking speed improvements with VR (alone or combined with conventional rehabilitation) compared to conventional therapy (moderate-to-very-low evidence) (23, 27), one study conversely found no intergroup differences (low evidence) (22). Notably, no significant benefits were observed in the 2-Minute Walk Test (very low evidence) (23).

##### 3.7.4.2 Intervention types

VR demonstrated superiority over no intervention (very low evidence). However, it showed no significant advantage over conventional rehabilitation alone, indicating comparable efficacy between modalities for ambulation improvement (28).

##### 3.7.4.3 Subgroup analyses

Subgroup analyses revealed that ambulation improvement was greater in the bilateral/mixed CP subtype compared to hemiplegia. Moreover, age and effect size exhibited a substantial negative linear relationship, with younger children exhibiting stronger therapeutic responses (29). These findings collectively suggest developmental-stage-dependent neuroplasticity and functional adaptability in pediatric populations. A limited number of SRs/MAs served as the basis for the subgroup analysis results presented above. This limitation may affect the generalizability and external validity of the findings, necessitating cautious interpretation.

#### 3.7.5 Improvement of ADL

Activities of daily living include the fundamental skills typically needed to manage basic physical needs (e.g., eating, dressing, personal hygiene, and toileting) and more complex activities related to independent living in the community (e.g., managing finances and medications) (42). Seven SRs/MAs evaluated VR interventions for ADL improvement in children with CP (23, 26, 31, 33–35, 37). The results of the GRADE evaluations for ADL are shown in Table 8.

##### 3.7.5.1 Assessment tools

Significant ADL improvements were observed using the Pediatric Evaluation of Disability Inventory and Canadian Occupational Performance Measure (low evidence) (35). However, conflicting results emerged from Functional Independence Measure for Children assessments: three SRs/MAs reported VR superiority (low evidence) (31, 35, 37), while one found no significant difference (very low evidence) (23).

##### 3.7.5.2 Intervention types

Isolated VR training demonstrated significant benefits over conventional rehabilitation (low evidence) (33). In contrast, combined VR-conventional approaches showed inconsistent efficacy, with no clear additive advantage observed (33).

##### 3.7.5.3 Subgroup analyses

Regarding system types, Kinect-based VR and depth-sensing motion capture systems outperformed digital imaging platforms in ADL outcomes (moderate evidence) (26, 31). Notably, non-immersive systems achieved greater functional gains compared to semi-immersive systems (37). For intervention parameters, optimal effects were achieved when sessions exceeded 30 min and cumulative exposure surpassed either 40 sessions or 1,200 min (26, 34). Specifically, task-specific VR protocols without combined conventional therapy yielded superior outcomes (moderate evidence) (26), indicating that focused VR training may better align with ADL-related neuroplastic adaptations. It should be noted that the subgroup analysis results described above were derived from a limited number of SRs/MAs, and careful interpretation is warranted.

### 3.8 Adverse reactions

Only two SRs/MAs reported adverse effects following VR treatment, with both indicating that no serious adverse effects were observed (28, 30).

## 4 Discussion

### 4.1 Results-based discussion

The methodological quality of the included SRs/MAs was predominantly rated as low or critically low according to the AMSTAR-2 assessment. A major concern was the frequent absence of *a priori* study protocols, which casts doubt on whether the analysis strictly followed predefined methodologies and increases the risk of bias. Many reviews also failed to justify their exclusive inclusion of RCTs, omitted lists of excluded studies, and did not report funding sources of the included trials. Additionally, most SRs/MAs did not adequately address the risk of bias in primary RCTs or sufficiently investigate sources of heterogeneity. The lack of sensitivity and subgroup analyses further undermined the reliability of the findings.

Evaluation using the PRISMA 2020 checklist revealed significant reporting deficiencies in the published SRs/MAs. Many studies did not disclose funding sources, raising concerns about potential conflicts of interest and publication bias. Transparency was further compromised by the absence of excluded study lists, preventing readers from assessing the rigor of study selection. Critical methodological details, such as protocol registration, were often missing, making it difficult to distinguish between preplanned and *post hoc* analyses. Furthermore, most reviews neglected to assess the quality of evidence for individual outcomes or employ statistical methods to detect publication bias, such as funnel plots or Egger/Begg tests.

The ROBIS tool identified several SRs/MAs with unclear or high risk of bias, primarily due to incomplete reporting of search strategies and study selection processes. Some reviews did not search clinical trial registries, potentially missing relevant studies, while others failed to document the number of reviewers involved in data extraction. A notable limitation was the lack of subgroup or sensitivity analyses to explore heterogeneity or test result stability. These shortcomings suggest that certain reviews may have overlooked key biases in their methodology, affecting the reliability of their conclusions.

According to the GRADE framework, the overall quality of evidence in the included SRs/MAs was mostly low or very low, with only a few exceptions rated as moderate. The primary reasons for downgrading included risk of bias in the original RCTs, particularly due to unclear randomization, allocation concealment, and blinding procedures. Substantial heterogeneity, often unexplained, further reduced confidence in the pooled results. Small sample sizes in many RCTs led to imprecise effect estimates with wide confidence intervals. Additionally, the inability to assess publication bias in reviews with few included studies weakened the overall evidence base.

While our PROSPERO protocol initially proposed using only AMSTAR-2 and GRADE for quality assessment, we expanded the evaluation to include PRISMA 2020 and ROBIS to ensure a more comprehensive appraisal. This decision was driven by the need to assess reporting transparency and domain-specific biases, which are not fully captured by AMSTAR-2 or GRADE alone. Although this deviation introduced additional analytical complexity, it significantly strengthened the validity of our findings. By integrating multiple assessment tools, we systematically identified common methodological limitations in published SRs/MAs, which may inform future research improvements and ultimately enhance the quality of evidence-based clinical decision-making.

### 4.2 Mechanisms of VR-based interventions in CP

#### 4.2.1 Motor learning and neuroplasticity

Motor rehabilitation approaches for cerebral palsy should be individualized, age and developmentally appropriate, goal-directed, skill-based, and intensive and time-limited (43). VR operationalizes core motor learning principles—including high-intensity repetition, progressive task difficulty, and multisensory feedback—to enhance neuroplasticity and skill acquisition (44). Neuroimaging studies have demonstrated that VR can significantly enhance the activation of brain regions associated with motor control, such as the primary motor cortex and supplementary motor area, indicating an increased responsiveness of the brain to motor tasks (45). Moreover, VR interventions also normalize aberrant neural activation patterns in CP, particularly by engaging the contralateral primary sensorimotor cortex, which correlates with improved functional motor skills (e.g., reaching, self-care activities) (46).

#### 4.2.2 Cognitive engagement and motivation

VR augments traditional rehabilitation paradigms by integrating cognitive-motor engagement during task execution, promoting neuroplasticity through combined motor repetition and problem-solving strategies (47, 48). The immersive environment imposes task-driven challenges that require continuous adaptive motor planning and error correction, reinforcing cognitive-motor strategy development in children with CP (49). Critically, VR’s gamified framework enhances treatment adherence through positive affective engagement, transforming repetitive exercises into goal-oriented activities. This dual focus sustains motivation during prolonged rehabilitation, potentially amplifying functional gains by increasing training tolerance and cortical activation (50).

#### 4.2.3 Sensorimotor integration

VR enhances sensorimotor integration in CP by improving visual perception, enabling precise body position and movement trajectory awareness, which optimizes motor accuracy and coordination (51, 52). Visual feedback within VR systems facilitates sensorimotor circuit reorganization and supports the development of compensatory behavioral strategies for functional deficits, critical for daily activity performance (53). Furthermore, VR environments allow safe, task-specific practice of complex motor patterns that are physically unfeasible in real-world settings, fostering neuroadaptive skill acquisition and functional independence (54).

#### 4.2.4 Postural control optimization

Research indicates that shifting attention from internal focus (movement accuracy) to external focus (task/goal-oriented stimuli) enhances balance acquisition and motor automaticity (55). VR facilitates this process by providing an immersive, task-oriented environment, directing children’s attention toward external goals, thereby specifically improving balance dysfunction and related motor control deficits (56). Unlike conventional therapy, VR frameworks embed purposeful tasks with multisensory feedback, prompting active engagement in hip flexion, abduction, and external rotation during standing exercises. This approach facilitates neurological recovery and balance improvement through task-specific kinematic reinforcement (57). Furthermore, VR training strengthens lower limb musculature, promoting symmetrical weight distribution and dynamic stability, which collectively enhance postural control and ambulatory capacity (58).

### 4.3 Interpretation of SRs/MAs results

The SRs/MAs included in this overview demonstrate that VR can improve the gross motor function of children with CP. Although VR has had a positive impact on balance, ambulation, ADL, and upper extremity motor function, not all SRs/MAs have reached positive conclusions. This discrepancy may be related to the different assessment tools used in the various SRs/MAs, the different RCTs included, and the varying quality of the studies. Additionally, the results of the meta-analyses are also affected by the size of the sample. Therefore, we need to interpret these results with caution.

It is essential to highlight that, among the 16 included SRs/MAs, only 10 SRs/MAs performed subgroup analyses (23–26, 28, 29, 31, 34, 36, 37). These subgroup analysis results were based on a limited number of SRs/MAs, necessitating a cautious approach to their interpretation. Subgroup analyses identified a significant negative linear association between participant age and VR efficacy (younger children showed greater functional improvements) and a positive dose–response relationship (higher intervention doses correlated with larger effect sizes) (29). These patterns may reflect heightened neuroplasticity in younger populations and the neurophysiological requirement for repetitive task-specific training to induce synaptic reorganization (59, 60). Discrepancies emerged regarding VR system efficacy: While some studies reported comparable outcomes between commercial gaming platforms and rehabilitation-specific systems (36), others demonstrated superior functional gains with engineer-developed, rehabilitation-tailored VR interfaces (29, 61). Customized systems allow precise calibration of task difficulty to individual functional levels and integration of co-design principles involving clinicians and patients, potentially enhancing therapeutic relevance and clinical adoption (62). Notably, non-immersive systems demonstrated greater ADL improvement than immersive systems in one study (37), possibly due to enhanced real-world skill transfer when maintaining environmental awareness (63). However, substantial heterogeneity, small sample sizes, and limited studies precluded definitive conclusions regarding critical moderators, including CP subtype, immersion level, and optimal intervention duration.

### 4.4 Study limitations

This overview has several methodological constraints. First, this overview only included published studies in Chinese and English, which may have omitted some studies conducted in other languages. Second, although the two reviewers received professional training and reached consensus on the assessment results through discussion or by consulting a more experienced third reviewer (Lihong Ma) when disagreements arose, some degree of evaluation bias may still exist, as quality assessment essentially relies on the judgment of the assessors. Third, outcome measures were limited to subjective scales, lacking complementary objective data. Fourth, significant heterogeneity across studies precluded meta-analytic synthesis, limiting consolidated efficacy interpretations. Fifth, recent RCTs not yet incorporated into existing SRs/MAs were excluded, narrowing the scope of evidence re-evaluation. Sixth, while restricting inclusion to RCT-focused SRs/MAs aimed to enhance quality, this approach excluded mixed-design reviews and may have underrepresented adverse events, potentially compromising risk–benefit assessments. Finally, the overall sample size greatly increased because many of the 16 SRs/MAs shared the same original studies. These results may have exaggerated the true efficacy and accuracy of VR interventions in the field of cerebral palsy. The differences between studies might not have been fully recognized because there were not enough truly independent studies, which could hide important variations and make the findings of this review less reliable and applicable.

### 4.5 Implications for further study

To handle substantial overlap among primary studies in systematic reviews, a two-step selection method is recommended. All non-overlapping systematic reviews should be included first. For overlapping reviews, priority should be assigned to Cochrane reviews, followed by an assessment of publication date, methodological quality, and relevance to the research question. Beyond addressing overlap, future SRs/MAs on VR interventions for CP should prioritize methodological rigor and transparency. Researchers are advised to prospectively register protocols on established platforms and adhere strictly to PRISMA 2020 guidelines to enhance reporting quality. Additionally, a comprehensive literature search strategy must be implemented, integrating database queries, reference screening, clinical trial registries, grey literature, and study registrations to minimize selection bias. Full-text exclusions should be explicitly documented with rationales to ensure reproducibility and mitigate reporting bias. When substantial heterogeneity is observed, subgroup or sensitivity analyses are warranted to explore underlying causes. Methodological limitations of included studies, including potential sources of bias and their implications for outcomes, must be critically discussed. Publication bias should be assessed using funnel plots alongside statistical tests to address small-study effects. Ultimately, the clinical relevance of findings should be supported by GRADE evaluations of critical outcome measures.

## 5 Conclusion

Current evidence suggests VR demonstrates therapeutic potential for improving upper limb function, gross motor skills, balance, ambulation, and ADL in children with CP, with no significant safety concerns reported. However, the robustness of these findings is compromised by the methodological limitations of the included SRs/MAs. Nevertheless, the cumulative evidence underscores VR as a viable adjunctive intervention for functional rehabilitation in this population. Further high-quality RCTs employing standardized protocols, objective biomarkers, and long-term follow-ups are imperative to validate efficacy, optimize implementation strategies, and strengthen translational relevance for clinical practice.

## Data Availability

The original contributions presented in the study are included in the article/Supplementary material, further inquiries can be directed to the corresponding author.

## References

[1] Sadowska M Sarecka-Hujar B Kopyta I. Cerebral palsy: current opinions on definition, epidemiology, risk factors, classification and treatment options. Neuropsychiatr Dis Treat. (2020) 16:1505–18. doi: 10.2147/ndt.S235165 32606703 PMC7297454

[2] Christine C Dolk H Platt MJ Colver A Prasauskiene A Krägeloh-Mann I . Recommendations from the SCPE collaborative group for defining and classifying cerebral palsy. Dev Med Child Neurol Suppl. (2007) 109:35–8. doi: 10.1111/j.1469-8749.2007.tb12626.x 17370480

[3] McGuire D Tian L Yeargin-Allsopp M Dowling N Christensen D. Prevalence of cerebral palsy, intellectual disability, hearing loss, and blindness, National Health Interview Survey, 2009-2016. Disabil Health J. (2019) 12:443–51. doi: 10.1016/j.dhjo.2019.01.005 30713095 PMC7605150

[4] Olusanya BO Kancherla V Shaheen A Ogbo FA Davis AC. Global and regional prevalence of disabilities among children and adolescents: analysis of findings from global health databases. Front Public Health. (2022) 10:977453. doi: 10.3389/fpubh.2022.977453 36249226 PMC9554924

[5] Demont A Gedda M Lager C De Lattre C Gary Y Keroulle E . Evidence-based, implementable motor rehabilitation guidelines for individuals with cerebral palsy. Neurology. (2022) 99:283–97. doi: 10.1212/WNL.0000000000200936 35750497

[6] Santer M Ring N Yardley L Geraghty A Wyke S. Treatment non-adherence in pediatric long-term medical conditions: systematic review and synthesis of qualitative studies of caregivers’ views. BMC Pediatr. (2014) 14:63. doi: 10.1186/1471-2431-14-63 24593304 PMC3984727

[7] Voinescu A Sui J Stanton FD. Virtual reality in neurorehabilitation: an umbrella review of Meta-analyses. J Clin Med. (2021) 10:1478. doi: 10.3390/jcm10071478 33918365 PMC8038192

[8] Abbas J O'Connor A Ganapathy E Isba R Payton T McGrath B . What is virtual reality? A healthcare-focused systematic review of definitions. Health Policy Techn. (2023) 12:100741. doi: 10.1016/j.hlpt.2023.100741

[9] Huygelier H Mattheus E Abeele VV van Ee R Gillebert CR. The use of the term virtual reality in post-stroke rehabilitation: a scoping review and commentary. Psychol Belg. (2021) 61:145–62. doi: 10.5334/pb.1033 34131490 PMC8176935

[10] Parsons T Riva G Parsons S Mantovani F Newbutt N Lin L . Virtual reality in pediatric psychology. Pediatrics. (2017) 140:S86–91. doi: 10.1542/peds.2016-1758I 29093039

[11] Palaus M Marron EM Viejo-Sobera R Redolar-Ripoll D. Neural basis of video gaming: a systematic review. Front Hum Neurosci. (2017) 11:248. doi: 10.3389/fnhum.2017.00248 28588464 PMC5438999

[12] Chen YP Kang LJ Chuang TY Doong JL Lee SJ Tsai MW . Use of virtual reality to improve upper-extremity control in children with cerebral palsy: a single-subject design. Phys Ther. (2007) 87:1441–57. doi: 10.2522/ptj.20060062 17895352

[13] Wille D Eng K Holper L Chevrier E Hauser Y Kiper D . Virtual reality-based paediatric interactive therapy system (PITS) for improvement of arm and hand function in children with motor impairment—a pilot study. Dev Neurorehabil. (2009) 12:44–52. doi: 10.1080/17518420902773117 19283533

[14] Huber M Rabin B Docan C Burdea GC AbdelBaky M Golomb MR. Feasibility of modified remotely monitored in-home gaming technology for improving hand function in adolescents with cerebral palsy. IEEE Trans Inf Technol Biomed. (2010) 14:526–34. doi: 10.1109/titb.2009.2038995 20071262

[15] Levac D Rivard L Missiuna C. Defining the active ingredients of interactive computer play interventions for children with neuromotor impairments: a scoping review. Res Dev Disabil. (2012) 33:214–23. doi: 10.1016/j.ridd.2011.09.007 22093667

[16] Pollock M Fernandes RM Becker LA. Chapter V: overviews of reviews In: Higgins JPT Thomas J Chandler J, editors. Cochrane handbook for systematic reviews of interventions. Version 6.1. London, UK: Cochrane (2020)

[17] Shea BJ Reeves BC Wells G Thuku M Hamel C Moran J . AMSTAR 2: a critical appraisal tool for systematic reviews that include randomised or non-randomised studies of healthcare interventions, or both. BMJ. (2017) 358:j4008. doi: 10.1136/bmj.j4008 28935701 PMC5833365

[18] Page MJ McKenzie JE Bossuyt PM Boutron I Hoffmann TC Mulrow CD . The PRISMA 2020 statement: an updated guideline for reporting systematic reviews. BMJ. (2021) 372:n71. doi: 10.1136/bmj.n71 33782057 PMC8005924

[19] Whiting P Savović J Higgins JP Caldwell DM Reeves BC Shea B . ROBIS: a new tool to assess risk of bias in systematic reviews was developed. J Clin Epidemiol. (2016) 69:225–34. doi: 10.1016/j.jclinepi.2015.06.005 26092286 PMC4687950

[20] Atkins D Best D Briss PA Eccles M Falck-Ytter Y Flottorp S . Grading quality of evidence and strength of recommendations. BMJ. (2004) 328:1490. doi: 10.1136/bmj.328.7454.1490 15205295 PMC428525

[21] Pieper D Antoine SL Mathes T Neugebauer EA Eikermann M. Systematic review finds overlapping reviews were not mentioned in every other overview. J Clin Epidemiol. (2014) 67:368–75. doi: 10.1016/j.jclinepi.2013.11.007 24581293

[22] Lin ST Luo MN Wang D Li ZK. Meta-analysis of the effects of telerehabilitation in patients with cerebral palsy. Chin Nurs Manag. (2019) 19:842–9. doi: 10.3969/j.issn.1672-1756.2019.06.009

[23] Luo TW Zhang N Tang Q Song QQ Xiang YQ Zhu LH. Effects of virtual reality technology on motor function of children with cerebral palsy:a Meta-analysis. Mil Nurs. (2023) 40:5–11. doi: 10.3969/j.issn.2097-1826.2023.07.002

[24] Ren Z Wu J. The effect of virtual reality games on the gross motor skills of children with cerebral palsy: a Meta-analysis of randomized controlled trials. Int J Environ Res Public Health. (2019) 16:3885. doi: 10.3390/ijerph16203885 31614990 PMC6843701

[25] Wu JL Loprinzi PD Ren ZB. The rehabilitative effects of virtual reality games on balance performance among children with cerebral palsy: a Meta-analysis of randomized controlled trials. Int J Environ Res Public Health. (2019) 16:4161. doi: 10.3390/ijerph16214161 31661938 PMC6861947

[26] Zhang Y Li R Miao X Cheng LJ Lau Y. Virtual motor training to improve the activities of daily living, hand grip, and gross motor function among children with cerebral palsy: Meta-regression analysis. Gait Posture. (2022) 91:297–305. doi: 10.1016/j.gaitpost.2021.10.046 34798421

[27] Han J Liang M Xie R. Meta analysis for effectiveness of virtual reality training on improving lower extremity function of children with cerebral palsy. Chin J Rehabil. (2020) 35:541–6. doi: 10.3870/zgkf.2020.10.009

[28] Fandim JV Saragiotto BT Porfírio GJM Santana RF. Effectiveness of virtual reality in children and young adults with cerebral palsy: a systematic review of randomized controlled trial. Braz J Phys Ther. (2021) 25:369–86. doi: 10.1016/j.bjpt.2020.11.003 33358737 PMC8353293

[29] Chen Y Fanchiang HD Howard A. Effectiveness of virtual reality in children with cerebral palsy: a systematic review and Meta-analysis of randomized controlled trials. Phys Ther. (2018) 98:63–77. doi: 10.1093/ptj/pzx107 29088476 PMC6692882

[30] Abdelhaleem N El Wahab MSA Elshennawy S. Effect of virtual reality on motor coordination in children with cerebral palsy: a systematic review and meta-analysis of randomized controlled trials. Egypt J Med Hum Genet. (2022) 23:71. doi: 10.1186/s43042-022-00258-0

[31] Liu C Wang X Chen R Zhang J. The effects of virtual reality training on balance, gross motor function, and daily living ability in children with cerebral palsy: systematic review and Meta-analysis. JMIR Serious Games. (2022) 10:e38972. doi: 10.2196/38972 36350683 PMC9685515

[32] Liu W Hu Y Li J Chang J. Effect of virtual reality on balance function in children with cerebral palsy: a systematic review and Meta-analysis. Front Public Health. (2022) 10:865474. doi: 10.3389/fpubh.2022.865474 35548088 PMC9081327

[33] Montoro-Cárdenas D Cortés-Pérez I Ibancos-Losada MD Zagalaz-Anula N Obrero-Gaitán E Osuna-Pérez MC. Nintendo® Wii therapy improves upper extremity motor function in children with cerebral palsy: a systematic review with Meta-analysis. Int J Environ Res Public Health. (2022) 19:12343. doi: 10.3390/ijerph191912343 36231643 PMC9566093

[34] Han Y Park S. Effectiveness of virtual reality on activities of daily living in children with cerebral palsy: a systematic review and meta-analysis. PeerJ. (2023) 11:e15964. doi: 10.7717/peerj.15964 37667752 PMC10475275

[35] Wang N Liu N Liu S Gao Y. Effects of nonimmersive virtual reality intervention on children with spastic cerebral palsy: a Meta-analysis and systematic review. Am J Phys Med Rehabil. (2023) 102:1130–8. doi: 10.1097/phm.0000000000002321 37535642

[36] Burin-Chu S Baillet H Leconte P Lejeune L Thouvarecq R Benguigui N. Effectiveness of virtual reality interventions of the upper limb in children and young adults with cerebral palsy: a systematic review with meta-analysis. Clin Rehabil. (2024) 38:15–33. doi: 10.1177/02692155231187858 37499213

[37] Komariah M Amirah S Abdurrahman MF Handimulya MFS Platini H Maulana S . Effectivity of virtual reality to improve balance, motor function, activities of daily living, and upper limb function in children with cerebral palsy: a systematic review and Meta-analysis. Ther Clin Risk Manag. (2024) 20:95–109. doi: 10.2147/tcrm.S432249 38375076 PMC10875340

[38] Calero-Morales S Del Consuelo V-BG Yance-Carvajal C Paguay-Balladares W. Gross motor development in preschoolers through Conductivist and constructivist physical recreational activities. Compar Res Sports. (2023) 11:61. doi: 10.3390/sports11030061 PMC10056820 36976947

[39] Fu T Zhang D Wang W Geng H Lv Y Shen R . Functional training focused on motor development enhances gross motor, physical fitness, and sensory integration in 5–6-year-old healthy Chinese children. Front Pediatr. (2022) 10:936799. doi: 10.3389/fped.2022.936799 35899135 PMC9309543

[40] Zhao H Deng Y Song G Zhu H Sun L Li H . Effects of 8 weeks of rhythmic physical activity on gross motor movements in 4-5-year-olds: a randomized controlled trial. J Exerc Sci Fit. (2024) 22:456–62. doi: 10.1016/j.jesf.2024.10.001 39502159 PMC11535996

[41] Zhou Y Tolmie A. Associations between gross and fine motor skills, physical activity, executive function, and academic achievement: longitudinal findings from the UK millennium cohort study. Brain Sci. (2024) 14:121. doi: 10.3390/brainsci14020121 38391696 PMC10887312

[42] Mlinac M Feng M. Assessment of activities of daily living, self-care, and Independence. Arch Clin Neuropsychol. (2016) 31:506–16. doi: 10.1093/arclin/acw049 27475282

[43] Faccioli S Pagliano E Ferrari A Maghini C Siani M Sgherri G . Evidence-based management and motor rehabilitation of cerebral palsy children and adolescents: a systematic review. Front Neurol. (2023) 14:1171224. doi: 10.3389/fneur.2023.1171224 37305763 PMC10248244

[44] Levin MF Demers M. Motor learning in neurological rehabilitation. Disabil Rehabil. (2021) 43:3445–53. doi: 10.1080/09638288.2020.1752317 32320305

[45] Golomb MR McDonald BC Warden SJ Yonkman J Saykin AJ Shirley B . In-home virtual reality videogame telerehabilitation in adolescents with hemiplegic cerebral palsy. Arch Phys Med Rehabil. (2010) 91:1–8. doi: 10.1016/j.apmr.2009.08.153 20103390

[46] You SH Jang SH Kim YH Kwon YH Barrow I Hallett M. Cortical reorganization induced by virtual reality therapy in a child with hemiparetic cerebral palsy. Dev Med Child Neurol. (2005) 47:628–35. doi: 10.1017/S0012162205001634 16138671

[47] Fu W Ji C. Application and effect of virtual reality Technology in Motor Skill Intervention for individuals with developmental disabilities: a systematic review. Int J Environ Res Public Health. (2023) 20:4619. doi: 10.3390/ijerph20054619 36901629 PMC10001794

[48] Doniger GM Beeri MS Bahar-Fuchs A Gottlieb A Tkachov A Kenan H . Virtual reality-based cognitive-motor training for middle-aged adults at high Alzheimer's disease risk: a randomized controlled trial. Alzheimers Dement. (2018) 4:118–29. doi: 10.1016/j.trci.2018.02.005 PMC6021455 29955655

[49] Deutsch JE Borbely M Filler J Huhn K Guarrera-Bowlby P. Use of a low-cost, commercially available gaming console (Wii) for rehabilitation of an adolescent with cerebral palsy. Phys Ther. (2008) 88:1196–207. doi: 10.2522/ptj.20080062 18689607

[50] Qian J McDonough DJ Gao Z. The effectiveness of virtual reality exercise on individual's physiological, psychological and rehabilitative outcomes: a systematic review. Int J Environ Res Public Health. (2020) 17:4133. doi: 10.3390/ijerph17114133 32531906 PMC7312871

[51] Bilde PE Kliim-Due M Rasmussen B Petersen LZ Petersen TH Nielsen JB. Individualized, home-based interactive training of cerebral palsy children delivered through the internet. BMC Neurol. (2011) 11:32. doi: 10.1186/1471-2377-11-32 21392370 PMC3061895

[52] Snider L Majnemer A Darsaklis V. Virtual reality as a therapeutic modality for children with cerebral palsy. Dev Neurorehabil. (2010) 13:120–8. doi: 10.3109/17518420903357753 20222773

[53] Chiang VC Lo KH Choi KS. Rehabilitation of activities of daily living in virtual environments with intuitive user interface and force feedback. Disabil Rehabil Assist Technol. (2017) 12:672–80. doi: 10.1080/17483107.2016.1218554 27782750

[54] Şahin S Köse B Aran OT Bahadır Ağce Z Kayıhan H. The effects of virtual reality on motor functions and daily life activities in unilateral spastic cerebral palsy: a single-blind randomized controlled trial. Games Health J. (2020) 9:45–52. doi: 10.1089/g4h.2019.0020 31335174

[55] Chiviacowsky S Wulf G Wally R. An external focus of attention enhances balance learning in older adults. Gait Posture. (2010) 32:572–5. doi: 10.1016/j.gaitpost.2010.08.004 20850325

[56] Mouhamed H Abo-Zaid N Khalifa H Ali M Elserty N Behiry M . Efficacy of virtual reality on balance impairment in ataxic cerebral palsy children: randomized controlled trial. Eur J Phys Rehabil Med. (2024) 60:949–55. doi: 10.23736/S1973-9087.24.08617-9 39441113 PMC11729712

[57] Lange BS Requejo P Flynn SM Rizzo AA Valero-Cuevas FJ Baker L . The potential of virtual reality and gaming to assist successful aging with disability. Phys Med Rehabil Clin N Am. (2010) 21:339–56. doi: 10.1016/j.pmr.2009.12.007 20494281

[58] Cho C Hwang W Hwang S Chung Y. Treadmill training with virtual reality improves gait, balance, and muscle strength in children with cerebral palsy. Tohoku J Exp Med. (2016) 238:213–8. doi: 10.1620/tjem.238.213 26947315

[59] Morgan C Novak I Badawi N. Enriched environments and motor outcomes in cerebral palsy: systematic review and meta-analysis. Pediatrics. (2013) 132:e735–46. doi: 10.1542/peds.2012-3985 23958771

[60] Gordon AM. Impaired voluntary movement control and its rehabilitation in cerebral palsy. Adv Exp Med Biol. (2016) 957:291–311. doi: 10.1007/978-3-319-47313-0_16 28035572

[61] Demers M Fung K Subramanian SK Lemay M Robert MT. Integration of motor learning principles into virtual reality interventions for individuals with cerebral palsy: systematic review. JMIR Serious Games. (2021) 9:e23822. doi: 10.2196/23822 33825690 PMC8060861

[62] Brassel S Power E Campbell A Brunner M Togher L. Recommendations for the design and implementation of virtual reality for acquired brain injury rehabilitation: systematic review. J Med Internet Res. (2021) 23:e26344. doi: 10.2196/26344 34328434 PMC8367177

[63] Perrochon A Borel B Istrate D Compagnat M Daviet JC. Exercise-based games interventions at home in individuals with a neurological disease: a systematic review and meta-analysis. Ann Phys Rehabil Med. (2019) 62:366–78. doi: 10.1016/j.rehab.2019.04.004 31078706

