# Supplementary material for: Effectiveness of virtual reality for functional disorders in cerebral palsy: an overview of systematic reviews and meta-analyses
Source: Front Neurol. 2025 Jul 30;16:1582110. doi: 10.3389/fneur.2025.1582110 (PMC12344382; doi:10.3389/fneur.2025.1582110)
Supplement: Supplementary file 1 [file Table_1.docx]

| #1 | (TI=(cerebral palsy OR brain palsy OR brain paralysis OR central palsy OR central paralysis OR cerebral paralysis OR cerebral paresis OR diplegia spastica OR encephalopathia infantilis OR palsy, cerebral OR spastic diplegia )) OR AB=(cerebral palsy OR brain palsy OR brain paralysis OR central palsy OR central paralysis OR cerebral paralysis OR cerebral paresis OR diplegia spastica OR encephalopathia infantilis OR palsy, cerebral OR spastic diplegia ) |
| --- | --- |
| #2 | (TI=(virtual reality OR Virtual rehabilitation OR virtual game OR virtual therapy OR virtual environment OR virtual technology OR Virtual Reality Exposure Therapy)) OR AB=(virtual reality OR Virtual rehabilitation OR virtual game OR virtual therapy OR virtual environment OR virtual technology OR Virtual Reality Exposure Therapy) |
| #3 | (TI=(Meta analysis as Topic OR meta-analysis OR systematic review OR systematic evaluation OR systematic assessment OR systematic overview)) OR AB=(Meta analysis as Topic OR meta-analysis OR systematic review OR systematic evaluation OR systematic assessment OR systematic overview) |
| #4 | #3 AND #2 AND #1 |

Web of Science

Embase

| #1 | ('cerebral palsy'/exp OR 'cerebral palsy') AND[embase]/lim |
| --- | --- |
| #2 | ('cerebral palsy':ti,ab,kw OR 'brain palsy':ti,ab,kw OR 'brain paralysis':ti,ab,kw OR 'central palsy':ti,ab,kw OR 'central paralysis':ti,ab,kw OR 'cerebral paralysis':ti,ab,kw OR 'cerebral paresis':ti,ab,kw OR 'diplegia spastica':ti,ab,kw OR 'encephalopathia infantilis':ti,ab,kw OR 'palsy, cerebral':ti,ab,kw OR 'spastic diplegia':ti,ab,kw) AND [embase]/lim |
| #3 | #1 OR #2 |
| #4 | ('virtual reality'/exp OR 'virtual reality') AND[embase]/lim |
| #5 | ('virtual reality':ti,ab,kw OR 'virtual rehabilitation':ti,ab,kw OR 'virtual game':ti,ab,kw OR 'virtual therapy':ti,ab,kw OR 'virtual environment':ti,ab,kw OR 'virtual technology':ti,ab,kw OR 'virtual reality exposure therapy':ti,ab,kw) AND [embase]/lim |
| #6 | #4 OR #5 |
| #7 | ('meta analysis':it OR 'systematic review':it OR 'meta analysis as topic':ti,ab,kw OR 'meta analysis':ti,ab,kw OR 'systematic review':ti,ab,kw OR 'systematic evaluation':ti,ab,kw OR 'systematic assessment':ti,ab,kw OR 'systematic overview':ti,ab,kw) AND [embase]/lim |
| #8 | #3 AND #6 AND #7 |

PubMed

| #1 | "Cerebral Palsy"[Mesh] |
| --- | --- |
| #2 | brain palsy[Title/Abstract] OR brain paralysis[Title/Abstract] OR central palsy[Title/Abstract] OR central paralysis[Title/Abstract] OR cerebral paralysis[Title/Abstract] OR cerebral paresis[Title/Abstract] OR diplegia spastica[Title/Abstract] OR encephalopathia infantilis[Title/Abstract] OR palsy, cerebral[Title/Abstract] OR spastic diplegia[Title/Abstract] OR cerebral palsy[Title/Abstract] |
| #3 | #1 OR #2 |
| #4 | ("Virtual Reality"[Mesh]) OR "Virtual Reality Exposure Therapy"[Mesh] |
| #5 | virtual reality[Title/Abstract] OR Virtual rehabilitation[Title/Abstract] OR virtual game[Title/Abstract] OR virtual therapy[Title/Abstract] OR virtual environment[Title/Abstract] OR virtual technology[Title/Abstract] OR Virtual Reality Exposure Therapy[Title/Abstract] |
| #6 | #4 OR #5 |
| #7 | (("Systematic Review" [Publication Type]) OR "Meta-Analysis" [Publication Type]) OR "Meta-Analysis as Topic"[Mesh] |
| #8 | Meta analysis as Topic[Title/Abstract] OR meta-analysis[Title/Abstract] OR systematic review[Title/Abstract] OR systematic evaluation[Title/Abstract] OR systematic assessment[Title/Abstract] OR systematic overview[Title/Abstract] |
| #9 | #7 OR #8 |
| #10 | #3 AND #6 AND #9 |

Cochrane library

| #1 | MeSH descriptor: [Cerebral Palsy] explode all trees |
| --- | --- |
| #2 | (cerebral palsy OR brain palsy OR brain paralysis OR central palsy OR central paralysis OR cerebral paralysis OR cerebral paresis OR diplegia spastica OR encephalopathia infantilis OR palsy, cerebral OR spastic diplegia):ti,ab,kw (Word variations have been searched) |
| #3 | #1 or #2 |
| #4 | MeSH descriptor: [Virtual Reality] explode all trees |
| #5 | (virtual reality OR Virtual rehabilitation OR virtual game OR virtual therapy OR virtual environment OR virtual technology OR Virtual Reality Exposure Therapy):ti,ab,kw (Word variations have been searched) |
| #6 | #4 or #5 |
| #7 | (meta-analysis):pt OR (systematic review):pt OR (Meta analysis as Topic OR meta-analysis OR systematic review OR systematic evaluation OR systematic assessment OR systematic overview):ti,ab,kw (Word variations have been searched) |
| #8 | #3 and #6 and #7 |

JBI

| #1 | (cerebral palsy or brain palsy or brain paralysis or central palsy or central paralysis or cerebral paralysis or cerebral paresis or diplegia spastica or encephalopathia infantilis or palsy, cerebral or spastic diplegia).ab, kw,ti. |
| --- | --- |
| #2 | (virtual reality or Virtual rehabilitation or virtual game or virtual therapy or virtual environment or virtual technology or Virtual Reality  Exposure Therapy).ab, kw,ti. |
| #3 | (Meta analysis as Topic or meta-analysis or systematic review or systematic evaluation or systematic assessment or systematic overview).ab,kw,ti,pt. |
| #4 | #1 and #2 and #3 |

CINAHL

| #1 | MH (MH "Cerebral Palsy") OR TI ( cerebral palsy OR brain palsy OR brain paralysis OR central palsy OR central paralysis OR cerebral paralysis OR cerebral paresis OR diplegia spastica OR encephalopathia infantilis OR palsy, cerebral OR spastic diplegia) OR AB( cerebral palsy OR brain palsy OR brain paralysis OR central palsy OR central paralysis OR cerebral paralysis OR cerebral paresis OR diplegia spastica OR encephalopathia infantilis OR palsy, cerebral OR spastic diplegia) |
| --- | --- |
| #2 | MH ( (MH "Virtual Reality") OR (MH "Virtual Reality Exposure Therapy") ) OR TI (virtual reality OR Virtual rehabilitation OR virtual game OR virtual therapy OR virtual environment OR virtual technology OR Virtual Reality Exposure Therapy ) OR AB ( virtual reality OR Virtual rehabilitation OR virtual game OR virtual therapy OR virtual environment OR virtual technology OR Virtual Reality Exposure Therapy ) |
| #3 | MH (MH "Meta Analvsis") |
| #4 | MH (MH "Systematic Review") |
| #5 | TI ( Meta analysis as Topic OR meta-analysis OR systematic review OR systematic evaluation OR systematic assessment OR systematic overview ) OR  AB ( Meta analysis as Topic OR meta-analysis OR systematic review OR systematic evaluation OR systematic assessment OR systematic overview) |
| #6 | #3 OR #4 OR #5 |
| #7 | #1 AND #2 AND #6 |

CNKI

| #1 | SU %= '脑瘫' OR SU %= '脑性瘫痪' |
| --- | --- |
| #2 | SU %= '虚拟现实' OR SU %= '虚拟环境' OR SU %= '虚拟康复' OR SU %= '虚拟游戏' OR SU %= '虚拟治疗' |
| #3 | SU %= '系统评价' OR SU %= 'Meta分析' OR SU %= '荟萃分析' OR SU %= '系统综述' OR SU %= '元分析' |
| #4 | #1 AND #2 AND #3 |

Wangfang

| #1 | 主题：脑瘫or脑性瘫痪 |
| --- | --- |
| #2 | 主题：虚拟现实or虚拟环境or虚拟康复or虚拟游戏or虚拟治疗 |
| #3 | 主题：系统评价or Meta分析or荟萃分析or系统综述or元分析 |
| #4 | #1 AND #2 AND #3 |

VIP

| #1 | 题目或关键词：脑瘫or脑性瘫痪 |
| --- | --- |
| #2 | 题目或关键词：虚拟现实or虚拟环境or虚拟康复or虚拟游戏or虚拟治疗 |
| #3 | 题目或关键词：系统评价or Meta分析or荟萃分析or系统综述or元分析 |
| #4 | #1 AND #2 AND #3 |

SinoMed

| #1 | [常用字段：智能]：脑瘫or脑性瘫痪 |
| --- | --- |
| #2 | [常用字段：智能]：虚拟现实or虚拟环境or虚拟康复or虚拟游戏or虚拟治疗 |
| #3 | [常用字段：智能]：系统评价or Meta分析or荟萃分析or系统综述or元分析 |
| #4 | #1 AND #2 AND #3 |
